# Impact of the Febrile Podcast and Learning Resource as an Infectious Diseases Education Platform

**DOI:** 10.1093/ofid/ofae124

**Published:** 2024-03-04

**Authors:** Sara W Dong, Wendy Stead

**Affiliations:** Division of Infectious Diseases, Departments of Medicine and Pediatrics, Emory University School of Medicine and Children's Healthcare of Atlanta, Atlanta, Georgia, USA; Division of Infectious Diseases, Beth Israel Deaconess Medical Center, Harvard Medical School, Boston, Massachusetts, USA

**Keywords:** digital medical education, Febrile, infographic, podcast, social media

## Abstract

**Background:**

Febrile is an infectious diseases (ID) podcast and learning platform with the aim of providing high-quality and accessible ID content for learners. We describe the use of Febrile as a resource for learning and teaching ID as well as learner satisfaction and perceived impact on clinical practice.

**Methods:**

The Febrile platform was launched in December 2020 and includes audio podcast episodes, infographics, and detailed online summaries of adult and pediatric ID topics. Production and contributor information is summarized. Podcast, website, and social media engagement is reported from available quantitative analytics. An online anonymous survey was conducted to assess educational impact.

**Results:**

After 3 years of operation, Febrile has produced 90 episodes and has been downloaded >460 000 times in 196 countries, with the majority of its audience (58.9%) listening from within the United States. A total of 230 participants from 30 countries and 38 US states completed the survey, of whom 79 (34.5%) were ID fellows in training and 78 (34.2%) were ID faculty physicians. Seventy-two percent of survey respondents reported visiting the website, and 82% had seen an infographic. Enhancing core ID knowledge was the primary driver for listening. Two-thirds of respondents indicated that information learned from Febrile has changed their practice, and 50% have used Febrile as a way to teach others. Febrile also led to favorable impressions of ID for those considering ID as a career.

**Conclusions:**

Febrile is an engaging platform for ID medical education and provides a unique resource within the global ID community.

## Teaser

The Febrile podcast and learning platform is an engaging educational tool for infectious diseases clinicians. Analytics demonstrate increasing use over the first 3 years, and survey results indicate its relevance to clinical practice and intangible benefits, such as community building.

## Graphical Abstract

**Graphical abstract:**
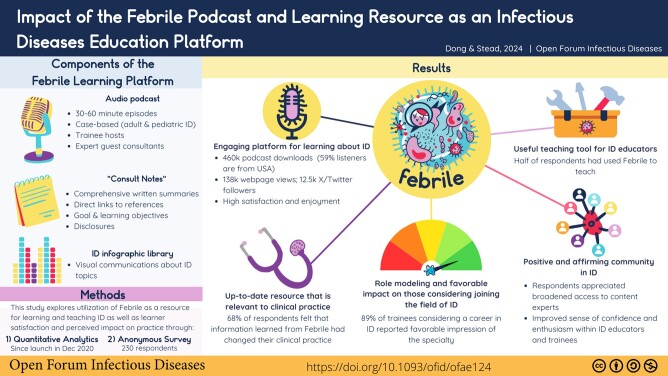

Digital medical education platforms can create robust online communities of practice that simultaneously deliver accessible and trusted educational content; foster a forum for interactive discussion; promote diversity, equity, and inclusion; and provide mentorship and sponsorship. Multimodal online platforms leverage the principles of self-directed learning, technology-enhanced learning, and various cognitive learning strategies to engage learners [1–3]. Multiple medical subspecialties have modeled this successfully, such as nephrology and cardiology [4–8]. Educational podcasts and their accompanying platforms in particular have grown into influential resources for medical trainees of all levels, physicians, and other clinicians. Surveys of US internal and emergency medicine residents showed that 60%–80% of trainees are using educational podcasts on a weekly or monthly basis, and more than a third of infectious diseases (ID) fellows are commonly using online resources like websites and podcasts at least once a week as complements to traditional teaching [9–11]. When asked what online-based resources they are most likely to use, 55% of ID fellows selected podcasts, a proportion that was exceeded only by access to online board questions (77%) [11]. Trainees have identified benefits such as ease of use, lower barrier to learning, an engaging format, broad content exposure, and the ability to foster connection to their peers and the larger professional community [11–13]. Podcasts have been similarly adopted by those beyond training and within other health disciplines as a flexible form of continuing medical education (CME) [4, 14].

The use and perceived impact of podcasts and other digital education resources is understudied despite the rapid growth in their use in medical education. Available literature has provided some initial insight into listening habits and possible motivations to incorporate digital resources into learning, but a deeper understanding is important for the development and improvement of future materials, particularly for the subspecialty learner. The current study aimed to explore how and why physicians and medical learners use Febrile, a medical podcast and learning platform, and investigate the perceived impact on learning and clinical practice.

Initially created as a resource for ID trainees, the Febrile platform is an integrated set of online multimedia resources available on a website (https://febrilepodcast.com/) and includes audio podcast episodes, visual infographics, and written summaries (blog posts). The mission statement of the Febrile podcast and learning platform is to provide a free open-access educational resource with high-quality content about ID. The podcast episodes use case-based learning and interviews with clinicians to discuss high-yield ID topics and model clinical reasoning. It was built to create a forum that connects the community of adult and pediatric ID colleagues and as a tool to showcase ID trainees, fellowships, and faculty around the country. The aspirational vision of Febrile is to ultimately inspire those in the future generation of ID physicians. Since Febrile is a free asynchronous public resource, listeners do not provide any identifying information or directed feedback. We present details on production of the platform resources, available analytics on the use of Febrile, and results from a survey aimed to assess current perceptions of the format, organization, and quality of the audio, written, and visual materials.

## METHODS

### Components and Production Process of the Febrile Learning Platform

The Febrile podcast and learning platform was launched on 28 December 2020 by author S. W. D. during her adult and pediatric (“Med-Peds”) ID fellowship training. She oversees production of all multimedia content, which includes selection, development, and review of topics and content; editing of contributor resources, including episode outlines and infographics; guest recruitment and scheduling; remote recording, audio editing, and final upload to media hosting; website creation and ongoing management. Audio podcast episodes are available for free in podcast directories, and all content is also available on the Febrile website (febrilepodcast.com).

Audio podcasts are generally produced every 1–2 weeks and aim to be 30–60 minutes in duration. Most episodes are structured as case-based discussions focused on diagnostic and/or management reasoning. This format includes one or two hosts who provide the clinical vignette or question prompts, along with one or two discussants. Contributors may be recruited based on topic expertise, or individuals can recommend guests or self-nominate for future episodes. S. W. D. meets with all contributors virtually to introduce the production process, answer questions, and assist with topic selection. Contributors are offered the opportunity to build a team from within their institution, which is a frequent model. If individual medical trainees reach out for involvement, they are also given the opportunity to use the episode process to network with an attending at a different institution, which is facilitated by S. W. D. If an individual faculty member expresses interest, they are encouraged to nominate or involve a trainee who might be interested.

Case-based scenarios are constructed for education or significantly altered and deidentified for learning purposes using best practices to protect patient privacy [15]. The episodes discuss both adult and pediatric ID content. Episode topics were initially selected with reference to available infectious disease board review blueprints or organically based on typical clinical consult questions encountered during S. W. D.'s fellowship training [16–19]. Listeners and/or guest contributors are able to provide suggested topics. Topics may also be selected for presentation in a “series” of related episodes. If topic content is anticipated to exceed the suggested episode duration, S. W. D. recommends that contributors split the material into multiple work products as a series.

The platform also includes supplementary materials. Each podcast episode is accompanied by “Consult Notes” on the website, which are comprehensive written supplements or blog posts available on the website (sometimes known as “show notes”; https://febrilepodcast.com/consult-notes/). These posts include the production team for an individual episode, summary of content from the episode, direct hyperlinks to references, goals and learning objectives, and disclosures. S. W. D. writes and edits the final form of all the Consult Notes, which are built from the contributing author's episode outline and clinical questions, material referenced during the audio podcast, and available related US guidance/guidelines or textbook resources. Audio transcripts are provided when possible. When available, transcription is created using an automatic artificial intelligence–powered service (Descript), followed by review by S. W. D. for corrections.

Podcast episodes are also generally paired with visual summary infographic as well, which are housed in an “infographic library,” available for review or download on the website and social media (https://sarawinndong.notion.site/Welcome-to-the-Febrile-ID-infographic-library-1c9af087a5314101a00bccacac0dd164). Febrile infographics commonly review content discussed in the audio episode and/or provide additional context and background related to the topic. The audio podcast does not come with the visual infographics attached, so individuals must independently encounter or seek out the infographics elsewhere. S. W. D. creates most of the infographics, and the remainder are created by other contributors with feedback. Final review before publication generally includes S. W. D. and the contributors involved for an episode. Infographic artists are cited directly on the visual aid as well as attributed with their infographic in the available library. S. W. D. typically uses an online graphic design application (Canva) as well as digital hand-drawn illustration (completed with use of the Procreate iPad app; Savage Interactive).

### Febrile Contributor Data

Full demographic information is not collected from contributors, but all participants provide a brief biography summarizing their current institution/location, level of training, and clinical or research interests. The effort and time commitment of creators involved in episode production is also not collected, but their role in production is categorized here by podcast writing (which includes editing or review), podcast guest, or infographic artist. If contributors were involved in 2 or more episodes, then their level of training was determined by their status at their first episode.

### Quantitative Analytics

Podcast analytics are available from the media host, Captivate (captivate.fm, owned by Captivate Audio Ltd), and are certified by the Interactive Advertising Bureau (iab.com), which sets a standard for download and listener behavior on the web and works with podcast hosting/analytics platforms to ensure unique downloads and listener counts meet industry standards. This study reports download statistics, including trends over time, geographic distribution of listeners, and listening platforms used. Website and X (formerly known as Twitter) engagement was obtained from Google Analytics 4 and Twitter analytics, respectively, to provide additional metrics.

### Survey Development

An online anonymous survey was created to assess educational impact, following best practices for questionnaires in educational research [20]. The objectives of the survey included collection of general demographic information, assessing the current satisfaction with and perceived value of various components of the platform (audio podcast, website and Consult Notes, and infographics), exploring motivations for listening, and acquiring feedback and recommendations for the future of the platform. The survey was conducted using Qualtrics. The survey was piloted with 8 individuals (including ID fellows, ID faculty physicians, and non-ID faculty) to evaluate the wording, layout, and response options. The final survey was generated with a combination of Likert scales, multiple choice, and open-ended response questions and is available in the Supplementary Materials. Respondents were provided the opportunity to add free-text comments on the impact of Febrile on perception of ID as a career, the strengths of Febrile platform, what could be improved with any of the Febrile platform materials, and suggestions for future episodes.

Any self-identified users of the Febrile learning resources (including listeners of the podcast and/or viewers of written or visual materials) were eligible to participate in the voluntary survey. No incentive was offered for participation. Recruitment requests for survey respondents were distributed via the audio podcast itself (verbal announcement of available survey with link in episode notes), on social media (X/Twitter, from the @febrilepodcast account), emails to current subscribers to the podcast, and ID-specific community message boards (Infectious Diseases Society of American Idea Exchange and Pediatric Infectious Diseases Society). Responses were collected from 22 January through 1 May 2022.

### Data Analysis

Descriptive data analysis is used to summarize contributor information and survey results. Open-text responses from the survey underwent qualitative thematic analysis, which was informed by principles of constructivist-grounded theory [21, 22]. Free text responses were coded using Excel, with an inductively developed and jointly agreed-upon codebook. Coding discrepancies were rectified before thematic analysis, with the overall goal of exploring how components of Febrile platform were used and perceived, motivations for use, and strengths, weaknesses, or recommended areas of improvement for Febrile. The Beth Israel Deaconess Medical Center Institutional Review Board determined the study exempt from the need for approval (protocol 2021P001060). This report does not include factors necessitating patient consent.

## RESULTS

### Febrile Platform Creators and Work Products

From December 2020 to December 2023, 90 podcast episodes were produced. The median audio episode length was 37 minutes 49 seconds (range, 4 minutes 13 seconds to 1 hour 11 minutes 57 seconds). Excluding the primary podcast host, S. W. D., there were 146 unique contributors to Febrile episodes over this 3-year period. Contributors included 7 medical students, 13 residents (United States/Canada), 9 registrars (United Kingdom), 28 ID fellows (United States/Canada), and 74 ID attending physicians. Other contributors included 4 non-ID attending physicians, 8 pharmacists, 2 microbiology specialists, and 1 nursing/infection control and prevention colleague. The majority of contributors specialized in the care of adults (97 of 146 [66.4%]). A quarter of respondents specialized in the care of children (36 of 146 [24.7%]) or were trained in both internal medicine and pediatrics (5 of 146 [3.4%]). The remainder did not identify a specific adult or pediatric clinical focus (8 of 146 [5.5%]). Over this period, 87 contributors were female (87 of 146 [60.0%]). Most contributors were based in North America (122 of 146 [83.6%]). Other regions represented included Europe (16 [11%]), Asia (6 [4.1%]), Australia (1 [0.7%]), and South America (1 [0.7%]). Participating contributors usually joined as discussants on the audio podcast (142 of 146 [97.2%]). Forty-eight contributors (32.9%) participated substantially in the writing of an episode, and 10 (6.9%) created an infographic. Twenty-two contributors were involved in more than 1 episode (22 of 146 [15.0%]). Details of contributors are available in Supplementary Table 1.

### Platform Use/Engagement Statistics

**Figure 1.**
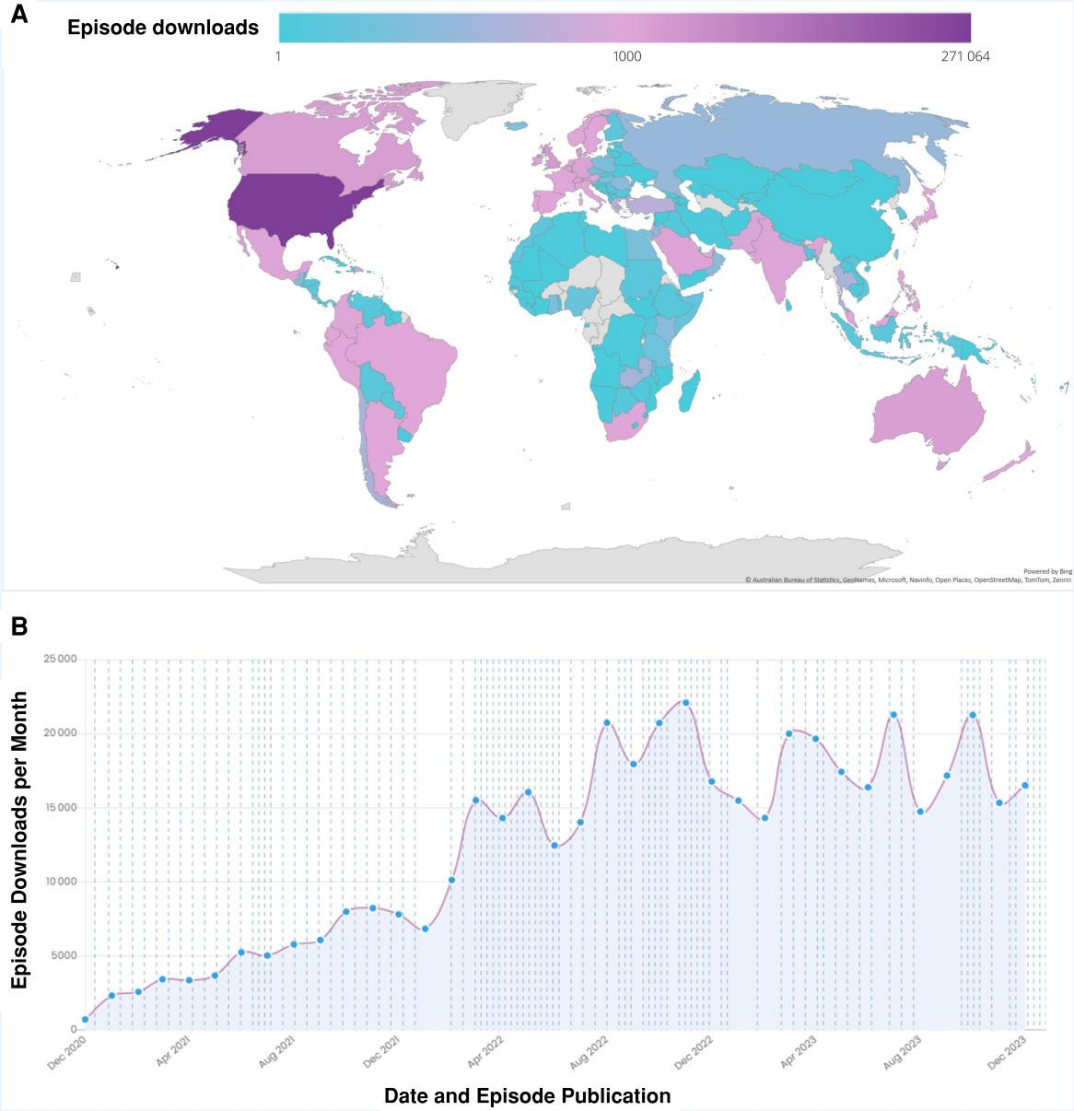
Podcast episode download distribution. *A,* Podcast episode download distribution by country. The 10 countries with the most total downloads were the United States (271 064 of 460 344 [58.9%]), the United Kingdom (30 096 [6.5%]), Canada (21 880 [4.8%]), Australia (19 617 [4.3%]), Germany (12 561 [2.7%]), Israel (8220 [1.8%]), Saudi Arabia (7915 [1.7%]), Mexico (6882 [1.5%]), India (5632 [1.2%]), and Ireland (5630 [1.2%]). *B,* Podcast episode downloads per month. Episode publication dates are overlaid with dotted lines.

In the first 3 years, Febrile had 460 344 unique downloads in 196 countries; Figure 1 represents downloads from 28 December 2020 through 31 December 2023. Most downloads are from the United States (271 064 [58.9%]), followed by the United Kingdom, Canada, Australia, Germany, and Israel. Listenership has grown since the initiation of the platform, with current individual episodes averaging more than 5000 downloads within 90 days of publication. The average total Febrile audio podcast downloads per month in 2023 was 17 473 per month (range, 14 324–21 292). Most listeners use a mobile app or browser to access the episodes (92.3%), such as Apple Podcasts (57.8%) and Spotify (18.2%). The febrilepodcast.com website had 138 000 page views from December 2020 through December 2023 (from Google Analytics 4 property data). X, previously known as Twitter, has garnered more than 12 500 followers.

### Survey Results

#### Respondent Demographics

A total of 230 participants from 30 countries and 38 US states completed the survey. Most respondents were from the United States (159 of 221 [71.9%]) and aged 26–35 years (130 of 229 [56.8%]). Approximately one-third of respondents were current ID fellows-in-training (79 of 228 [34.7%]) and were largely from adult ID training programs (56 of 79 [70.9%]). The next largest groups represented included ID faculty physicians (78 of 228 [34.2%]), medical residents or registrars (24 of 228 [10.5%]), and pharmacists (11 of 228 [4.8%]). Most respondents described their practice setting as academic/university hospital affiliated (170 of 227 [74.9%]). Full details on demographic responses are noted in Table 1 and Supplementary Table 2.

**Table 1.** Survey Respondents’ Demographic Information and Reported Use of Febrile Learning Platform

Question		No./Total Respondents (%)^a^
Age, y	≤25	8/229 (3.49)
	26–35	130/229 (56.77)
	36–45	64/229 (27.95)
	46–55	16/229 (6.99)
	≥56	11/229 (4.80)
Are you an ID fellow-in-training?	Yes	79/228 (34.65)
Are you an adult and/or pediatric ID fellow?	Adult ID fellow	56/79 (70.89)
	Pediatric ID fellow	19/79 (24.05)
	Combined adult and pediatric fellow	4/79 (5.06)
What is your current year of fellowship training?	First	28/78 (35.90)
	Second	24/78 (30.77)
	Third	16/78 (20.51)
	Fourth or more	10/78 (12.82)
What is your professional role?	Medical student	7/149 (4.70)
	Other type of student	0/149 (0.00)
	Medical resident or registrar	24/149 (16.11)
	Fellow-in-training, non-ID	0/149 (0.00)
	Faculty physician, ID^b^	78/149 (52.35)
	Faculty physician, non-ID^c^	10/149 (6.71)
	Pharmacist	11/149 (7.38)
	Pharmacy resident^d^	3/149 (2.01)
	Microbiologist or laboratory technician	9/149 (6.04)
	Advanced practice provider (APP) or APP student	4/149 (2.68)
	Nurse	0/149 (0.00)
	Other healthcare professional	3/149 (2.01)
	I am not a healthcare professional	0/149 (0.00)
For medical students: What year of medical school?	First	0/7 (0.00)
	Second	0/7 (0.00)
	Third	3/7 (42.86)
	Fourth or more	4/7 (57.14)
For faculty physicians: How many years have you been in practice?	<1	15/86 (17.44)
	1–2	14/86 (16.28)
	3–5	12/86 (13.95)
	>5	45/86 (52.33)
What is your practice setting?	Academic	170/227 (74.89)
	Community	43/227 (18.94)
	Industry	0/227 (0.00)
	Military	5/227 (2.20)
	Other	9/227 (3.96)
How many episodes of the Febrile podcast have you listened to?^e^	≤10	86/230 (37.39)
	11–20	87/230 (37.83)
	≥21	57/230 (24.78)
Do you ever listen to an episode more than once?	Yes	118/230 (51.30)
Have you visited the febrilepodcast.com website?	Yes	167/230 (72.61)
How many times have you accessed the website?	1–2	32/167 (19.16)
	3–5	60/167 (35.93)
	>5	75/167 (44.91)
Did you look at the “Consult Notes” for an episode?	Yes	143/167 (85.63)
Have you seen a Febrile podcast related infographic?	Yes	187/229 (81.66)
Where have you seen Febrile infographics?	On Twitter	156/175 (89.14)
	On Instagram	20/143 (13.99)
	On the febrilepodcast.com website	132/176 (75.00)
	Used in teaching materials I saw	45/147 (30.61)
	Other^f^	2/122 (1.64)

Abbreviation: ID, infectious diseases.

^a^Not all participants provided a response for all questions, leading to some variability in survey denominators in certain questions.

^b^Responses on specialization within ID: adult (n = 61), pediatric (n = 13), and adult and pediatric (n = 3).

^c^When asked for specialty, responses included internal medicine or hospital medicine (n = 17), emergency medicine (n = 2), pediatrics (n = 2), internal medicine–pediatrics (n = 4), and dermatology, family medicine, clinical virology (n = 1 each).

^d^All pharmacy trainee respondents were postgraduate year 2 residents.

^e^During the survey period of January through May 2022, Febrile released episodes 29–44.

^f^During the ID Fellows Cup game [23].

#### Engagement With Platform Materials

Approximately half of respondents described listening to an episode more than once (118 of 230 [51.3%]). Most respondents had visited the febrilepodcast.com website before (167 of 230 [72.6%]) and had accessed the website multiple times. The Consult Notes specifically were frequently viewed (by 143 of 167 [85.6%]). When queried about infographics, the majority had seen a Febrile-related infographic (187 of 229 [81.7%]). These infographics were most frequently seen on X/Twitter and the Febrile website, although respondents also interacted with infographics in other formats, including prepared teaching materials.

#### Respondent Satisfaction With Febrile

**Figure 2.**
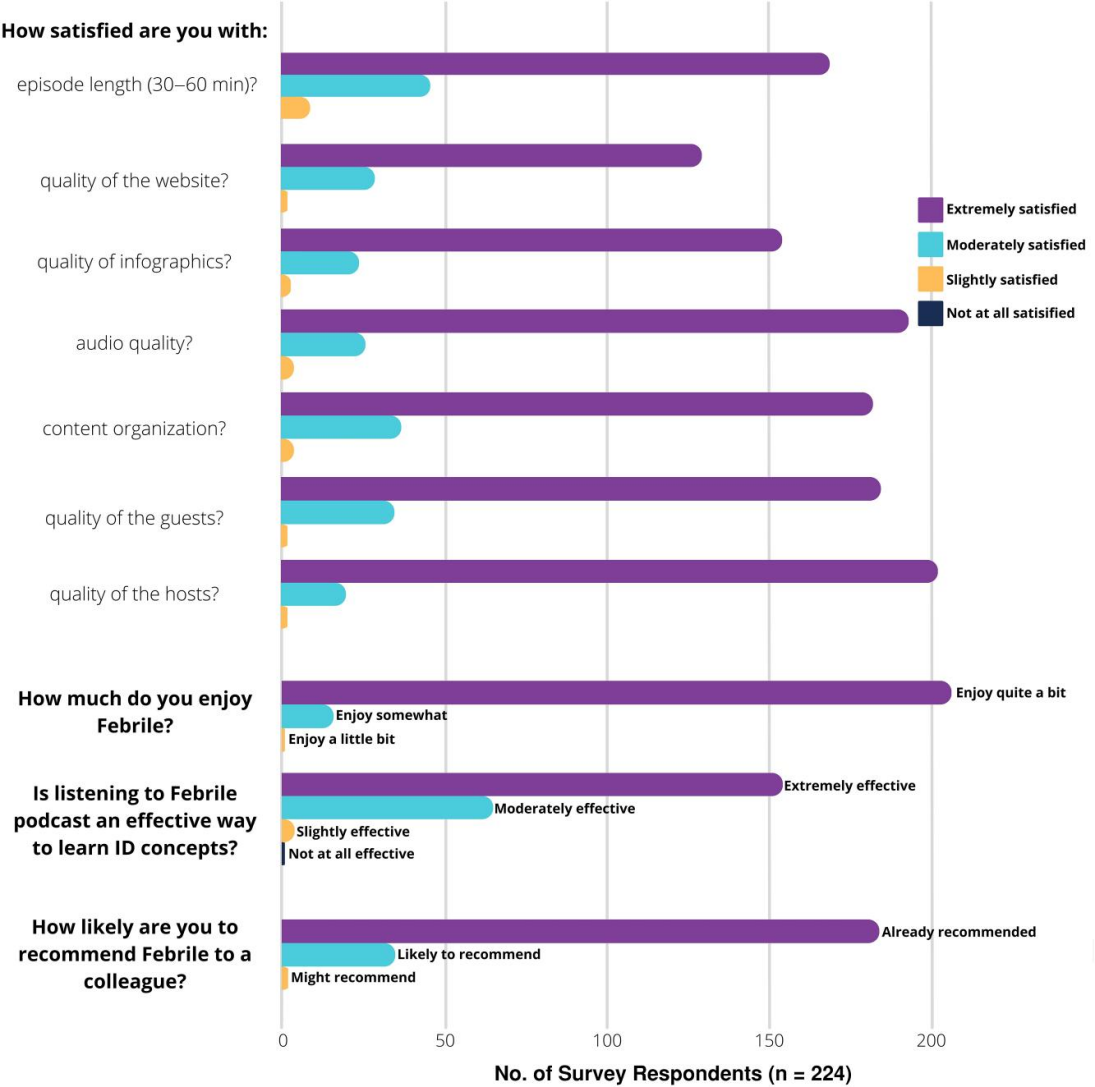
Reported satisfaction with Febrile from survey respondents. Results are also available in Supplementary Table 3. Abbreviation: ID, infectious diseases.

Most respondents were extremely or moderately satisfied with features or components of the platform, including episode length, content organization, and audio quality, as well as the quality of the website, infographics, guests, and hosts (Figure 2, Supplementary Table 3). The majority selected that listening to Febrile podcast episodes was a moderately or extremely effective way to learn ID concepts (67 of 230 [29.1%] and 158 of 230 [68.7%], respectively). Respondents also described enjoying Febrile (“quite a bit” 212 of 229 [92.6%]) and frequently recommended the resource to a colleague (190 of 227 [83.7%]).

#### Perceived Impact and Motivations for Use of Platform

**Figure 3.**
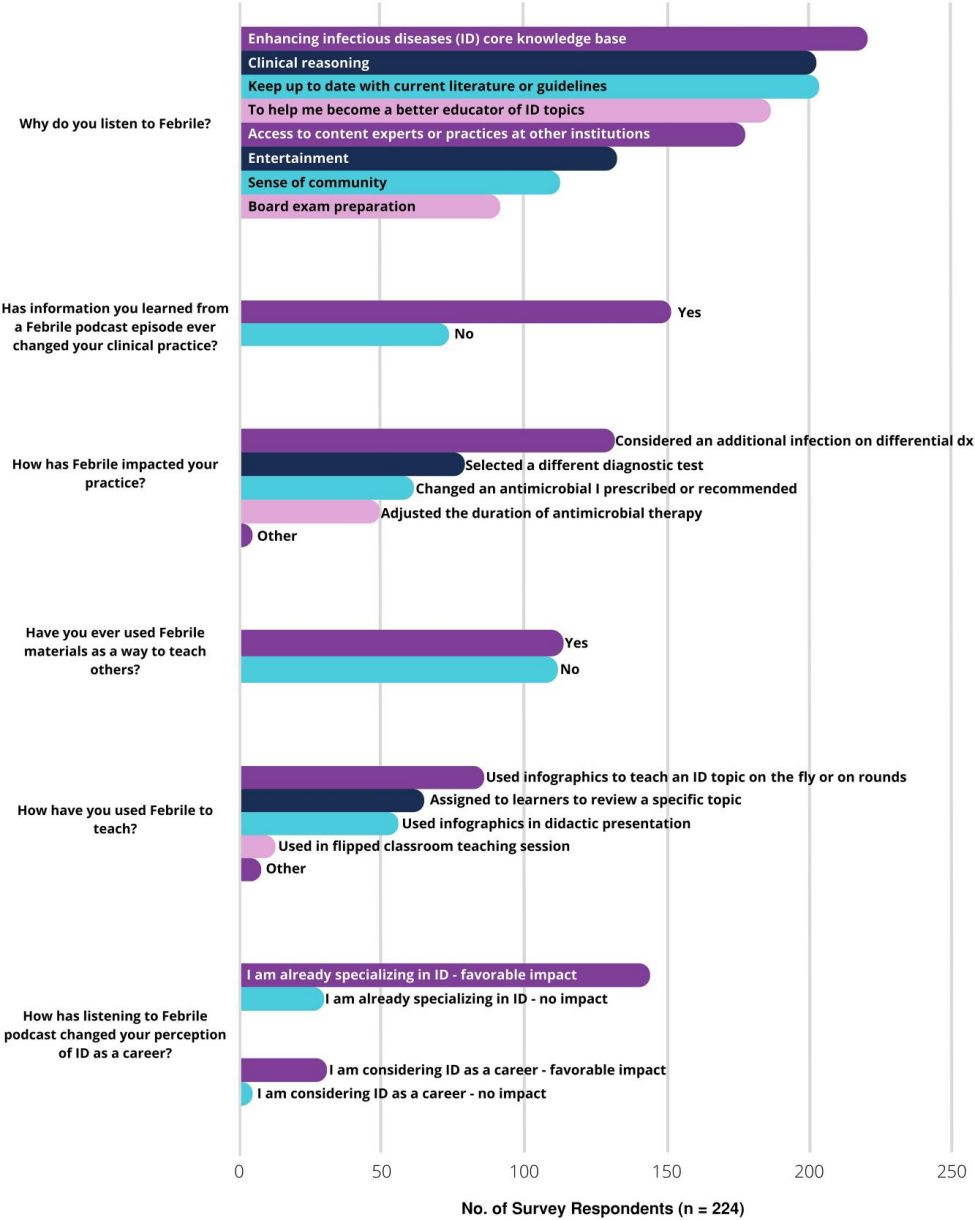
Perceived value and motivations for use of Febrile. Results are also available in Supplementary Table 4. Abbreviation: dx, diagnosis.

Multiple reasons for listening to Febrile were selected, particularly to enhance the ID core knowledge base, clinical reasoning, and to become a better educator on ID topics (Figure 3 and Supplementary Table 4). When asked whether information learned from a Febrile podcast episode had ever changed their clinical practice, 68.3% (157 of 230) answered yes. This was typically related to consideration of an additional infection on their differential diagnosis (137 of 150 [91.3%]) or selecting a different diagnostic test (84 of 136 [61.8%]). Other selected adjustments in practice included changing an antimicrobial prescribed or recommended (65 of 135 [48.2%]) and adjusting the duration of antimicrobial therapy (52 of 133 [39.1%]). Listening to Febrile had a favorable impact on those already specializing in ID (148 of 177 [83.62%]), as well as those considering a career in ID (31 of 35 [88.57%]).

#### Use of Febrile as a Teaching Tool

Half of respondents have used Febrile materials to teach others (117 of 230 [50.9%]). Methods of teaching included use of infographics to teach an ID topic on the fly or on rounds (78.4%) or to prepare a didactic presentation (54.3%), assignment of the podcast to learners to review a specific topic (60.9%), and use of a podcast in flipped classroom teaching session (13.0%).

### Qualitative Analysis

Thematic analysis of the free-text comments in the survey yielded 6 themes with respect to Febrile usage, strengths, and areas for improvement. Themes are presented below and summarized with representative quotes in Table 2.

**Table 2.** Themes and Representative Respondent Quotes

Theme	Representative Quotes^a^
Febrile as a relevant clinical resource	I love the idea. Every podcast has a broad list of important articles that I try to read afterwards to become more familiar with the topic as I’m in the first year of ID fellowship so my knowledge is still very limited. For example, I did not realize that rifampin use for staph PJI was based on such a small trial and that there are new articles published recently that question validity of the results. A clinical ID podcast was greatly needed and this does a great job in filling it. I appreciate the variety of ID educators across different institutions. Love this podcast. Great way to review topics and remind practitioners (myself included) to stay up to date and evidence based instead of being biased by anecdotes. Relevant topics are discussed. I enjoy the case-based format. The differentials and clinical decision making presented by the guests are top notch. I enjoy the “bit of culture” section, as it keeps it a bit more casual than other ID podcasts which feel too “protocolar.” It is also a plus that it does not feel political or commercially influenced, like unfortunately some other ID podcasts currently do. Really a fantastic project overall that I have found extremely enjoyable and also useful for my practice.
Quality of Febrile platform components	Febrile is the best!!! The content is great, the host has excellent podcast presence, the website is wonderful. I want to make sure it’s a sustainable part of the ID community. The info graphics and consult notes are the best “show notes” I have seen from a podcast. Overall [a] strong positive impact. The case presentations, quality of organization and questions developed, and quality of speakers answering is superb! Host is excellent. The website is also a great resource High quality content, expert guests, hits on key clinical topics, infographics incredibly useful, well researched consult notes Really high quality discussions which are entertaining and enthusiastic. Fun and informative, emphasis on clinical reasoning in infectious diseases, offers a breadth of topics and guests, excellent infographics and show notes that I often use as teaching materials, creating an online community for ID physicians and educators, fostering curiosity.
Technology-enhanced learning	To use my commute time effectively to stay engaged and learn/stay fresh. Good content, portable (easy to listen to when getting ready in the mornings or on my drive to work), beautiful, easy to read and info-rich graphics. Easily digestible source of relevant knowledge that can be coupled with other activities for busy lives (like when walking my dog). Variety of topics, detailed discussions, multiple different ways of presenting the material.
Community in ID	Absolutely fabulous! Keep it up, I love it. It inspires me to keep going despite being in an environment where no one respects ID and I will soon be the only one left at hospital. Fun, ID-community building, infographics, case based discussion. Number one strength: it gives current and aspiring ID peeps a sense of larger community. You couldn’t have launched this at a better time (aka during the pandemic)! It's positive and affirming. I needed that, especially as ID feels undervalued, underappreciated and lonely. It is just inspiring, I already knew several concepts, but just listening for remember, laugh and see the community spirit in COVID times, was priceless. I have had a lot of time out of training to have babies and do research. These podcasts have really helped refresh my memory and improved my confidence for returning to work.
Careers in ID	Good to hear the various different career paths that the guests have taken. I discovered Febrile at the recommendation of a classmate last year. I am an incoming med-peds ID fellow and was so excited to discover that Sara is also a med-peds ID fellow! I love that you mention that at the beginning of each conference because I don't think many med students, or even some residents, realize a combined fellowship is a career option! The Febrile podcast showcases how ID physicians are not only specialists, but also excellent general internists and in turn, excellent diagnosticians. The podcast sparks curiosity in a multitude of areas of infectious diseases and also showcases how different experts think through these complicated ID cases. I feel energized and inspired after listening to a podcast episode and this only further confirms my passion for the field of ID and as a career.
Febrile areas for improvement	Since the puscast ended there’s an opportunity to expand and review the current literature. You’re starting to do this with “digest” and I would encourage you to expand and build on that. It would draw in a larger, older/attending level crowd. Consider having an academic expert and a community-based ID doc on to talk about the same subject and how their approaches might vary. Excellent resource, very well done. Also accessed internationally (I'm in the UK) so would like to see reach beyond US practice once it inevitably expands much further. Including experts from other countries (throughout the world) would be an excellent addition. It would be nice to have fundamentals podcasts for those not ID fellows. I work in EM and it would be great to hear your approach to challenging infectious situations we see ourselves in. Also would be helpful to hear areas we screw up from your perspective. Some of the didactic portions from guests get a little “long in the tooth.” Suspect this is because of high interest of the guest, but it can also be hard to focus on core concepts. Perhaps breaking down 3–5-min speaking sections by guests into more of back and forth with hosts.

Abbreviations: COVID, coronavirus disease 2019; EM, emergency medicine; ID, infectious diseases; PJI, prosthetic joint infection.

^a^Quotes are provided verbatim and in their entirety from participant survey open-answer entries.

#### Febrile as a Relevant Clinical Resource

The use of a clinical case format with modeling of clinical reasoning was valued as a strength of Febrile. The clinical cases and interview questions were described as well organized and systematic. The content was also recognized for the ability to simultaneously engage multiple levels of learner, with one describing Febrile as “Accessible and pitched at the right level for both trainees and established physicians, which is hard to achieve.” Respondents described the content covered as relevant to their daily clinical practice and enjoyed the diversity of both topics and guests. These characteristics were also connected to the use of Febrile as a reference for further personal self-directed learning or as recommendation to colleagues and/or trainees.

#### Quality of Febrile Platform Components

Respondents echoed their satisfaction with the Febrile platform in the free-text responses, with a particular emphasis on the detail and quality of the written and visual materials. The website and Consult Notes were described as accurate, comprehensive, and easy to navigate. The infographics were frequently cited as a unique strength for the podcast, and these were found to be high quality, well designed, and useful for personal or teaching reference. Interactive discussions from the audio podcasts were described as informative but also fun, entertaining, and collegial. Multiple people also commented on the high production value with good audio quality.

#### Technology-Enhanced Learning

The digital platform was useful for users, especially the ease of access and ability to multitask. Febrile users shared that the platform benefited from the multimodal learning experience, and some suggested the possible addition of short educational videos. The podcast, infographic, and website materials were described as easy to digest and concise, as summarized by one respondent with “consolidates maximum up-to-date knowledge on the subject in a limited time.” The ability to quickly identify links to additional trusted references for further study was a benefit, with multiple respondents adding how they accessed important resources from the Consult Notes.

#### Community in ID

Survey respondents appreciated the ability to gain insight and perspectives from others in the ID community and believed that the platform allowed for broadened access to other colleagues they may not have otherwise encountered. The “expert” approach was highly valued. For example, one person wrote “I love that you pull expert opinions from institutions all over the country! It is great to learn the perspective of so many incredible ID physicians.” The platform was noted to provide a sense of community spirit or support among those in ID, particularly in contrast to feelings of being undervalued or isolated in their local setting. This sense of support was linked with expressions of increased confidence, enthusiasm, or inspiration. Many respondents expressed the hope to keep the Febrile resource running and sustainable so that it would remain a resource to the ID community.

#### Careers in ID

Respondents commented on the ability of Febrile to spark curiosity or excitement about a career in ID, and this was also described in reference to interactions with trainees, such as “love the energy and enthusiasm this brings to residents.” Other responses described the indirect career role modeling that can be provided by featuring guests with a diversity of career paths. Other respondents shared how Febrile was a confirming resource for their desire to pursue ID, such as “Makes me 100× more sure that I want to do ID!!!”

#### Febrile Areas for Improvement

Areas for possible growth noted by respondents included reviews of recent ID literature and expanding guest perspectives, including voices from those who practice in more community or rural settings and internationally. There were variable responses on the appropriate level of medical knowledge to target. Some asked for more complexity for the ID specialist (“I would like more specifics on the complications of treatment. ID gets difficult when things go wrong [eg, specifics of liver failure on TB treatment]”), while others suggested providing more fundamental materials on ID topics that target the non-ID audience, such as those in the hospital or in emergency medicine. Many endorsed the use of short-duration episodes, with specific requests for episodes under 20–30 minutes. Respondents hoped for availability of comprehension questions and/or CME credit in the future. From a technology perspective, respondents suggested an app to accompany the learning platform, an ability to search the website and infographic library more easily, and consistency in audio quality across guests.

In response to these survey responses, some changes were previously incorporated into the platform in 2022–2023. Additional shorter formats and styles of episodes were trialed, and the online infographic library was rebuilt to now be easily searched by keyword and topic.

## DISCUSSION

This study explored how and why individuals use a specific infectious disease podcast and learning platform, Febrile, by reporting podcast and web-based analytics along with survey results. The Febrile platform has been enthusiastically received by our ID community, as evidenced by the growing use noted in available quantitative analytics and survey responses regarding perceptions of the platform. The podcast is nearing half a million downloads after 3 years of production with a wide international reach. Respondents shared their satisfaction with Febrile materials, and several themes were identified that suggest Febrile is a high-quality resource that provides tools for both learning and teaching clinical ID knowledge while also creating a sense of community for ID clinicians.

Febrile serves as both a learning and teaching tool for ID trainees and educators that leverages the features of technology-enhanced learning. A key strength of Febrile noted in this study is the focus on clinically relevant and up-to-date ID content. The clinical case format organization provides a way to gain insight from content experts and master clinicians through informative and engaging discussions. Survey respondents enjoyed this expert perspective and modeling of diagnostic and management reasoning, which was believed to represent a unique offering in the ID digital resource landscape. Topics were seen as relevant to daily clinical practice, and Febrile materials were frequently used for both self-reference and recommendations to trainees or colleagues. Whether the podcast could truly cause a change in behavior was not assessed in this study, but 68% of survey respondents did perceive that the knowledge they learned from Febrile affected their clinical practice in a meaningful way. Respondents also confirmed their use of Febrile multimedia materials to teach others, and Febrile infographics in particular were highlighted by respondents as useful and high-quality visual accompaniments that summarized complex information. Infographics were used to teach on the fly or during rounds in most cases, but other methods, such as inclusion in didactic materials, were described as well. Febrile has been noted in previous publications as a well-received multimedia project for ID learners [1, 11, 24].

Community building in ID is an important part of the Febrile mission and was identified as a theme in survey responses. Access to the voices of experts in ID was highly valued, and respondents also described feelings of excitement, enthusiasm, and encouragement about being a member in the field of ID. The ability for online communities of practice and digital education platforms to provide a place for personal and professional development has been appreciated in similar projects outside ID [5, 8, 25]. In this study, we also share the crucial involvement of the ID community in this initial period of Febrile. It is important to emphasize that episodes are made possible by the donated effort and time of many guest contributors, including more than 50 trainees and more than 75 other medical educators who participated in content creation over the first 3 years. Febrile provides a platform for trainees and junior faculty in our ID community to expand their educational reach to this international audience and often allows more in-depth and nuanced reviews of topics compared with typical social media or microblogging interactions. A more detailed exploration of the experience of this hands-on participation for contributors would be a valuable area of future study. Creation of digital education resources is an important skill set that modern medical educators require, and this need has prompted the development of innovative new programs and curricula to cultivate these skills, such as the Nephrology Social Media Collective Internship, CardioNerds Academy, and the ID Digital Institute [26–28].

Another notable feature of this study was the signal that Febrile may lead to a favorable impression of ID careers to those contemplating joining the field. Although trainees were a smaller portion of the survey respondents, 89% reported a positive impression of a career in ID from listening to Febrile on the directed survey question, and several free-text comments validated this excitement about pursuing a career path in ID. Role modeling and early exposure to ID plays a key role in recruitment, and Febrile may serve as another tool available to introduce or expand exposure to ID for trainees [29, 30]. Given recent challenges matching applicants to both adult and pediatric ID fellowship training, the importance of innovative resources that may attract prospective trainees to the field by using media they routinely interact with cannot be overstated [31].

Digital education tools such as Febrile do have disadvantages. Peer review processes are not as rigorous as traditional scholarship, although this is somewhat offset by careful review of individual episodes and informal subsequent community review. Quality evaluation tools for blogs and podcasts used in medical education are available, such as the revised METRIQ score [32, 33]. These tools emphasize features that the Febrile podcast and Consult Notes strive to provide, such as mapping specific references to statements and supplying clear identity, qualifications, and conflicts of interests of authors. Febrile would benefit from expanded assistance in the editorial and review process of materials, although finding dedicated longitudinal partners is challenging for an unfunded project. This study also elicited specific areas for improvement for the platform, with respondents noting ID literature reviews, additional topics and guests, and availability of comprehension questions for future content.

There are limitations to this study, and it remains challenging to truly quantify uptake and the impact of a digital resource such as Febrile. Downloads do not necessarily represent actual listening completed by the learner and cannot be equated to comprehension. The response rate for the survey cannot be calculated, given the unclear denominator of unique individuals who either listen to the podcast or use other Febrile resources. There is evolving literature on how to best assess digital scholarship efforts and define their impact, particularly in the context of promotion and tenure for individual creators, but no broadly accepted standard exists. One example suggests that the metrics of >5000 and >20 000 downloads per month over a calendar year should be classified as medium and high impact, respectively, for an editor-in-chief of an academic or clinical podcast, which would classify Febrile as a moderate- to high-impact educational intervention [34]. Although efforts were made to seek out any audience of Febrile, this survey recruitment approach still may bias responses to those who preferentially use online resources. However, this study still provides unique insight into motivations for using this resource and was able to highlight specific areas of future improvement from the population that likely uses Febrile the most. The study's analysis was limited to free-text survey responses, but future use of focus group interviews with qualitative analysis would provide more extensive insight into the themes or key factors influencing podcast usage and the user experience. Future studies could also consider evaluating episode learning objectives with specific knowledge evaluations.

Future hopes for the Febrile platform include continued expansion and innovation in CME opportunities, in particular, availability of comprehension questions, debate-style sessions, and focused resources for early trainees, advanced practice providers, and, possibly, non-ID clinicians. Additional anticipated projects already in motion include Spanish-language episodes and reinforcement of core ID topics by highlighting relevant review articles or clinical cases published in Infectious Diseases Society of America journals. Febrile hopes to continue exploring ways to partner with other digital ID resources (such as other podcasts, ID Fellows Cup, #IDJClub, the ID Digital Institute, and the ID Fellows Network), individual fellowship programs, local or national ID societies, and affiliated journals to provide cross-platform synergy. The best way to integrate podcast resources into local rotation education or curriculums is also an area ripe for study.

In conclusion, the Febrile podcast and learning platform is an engaging and high-quality tool for creating and disseminating ID knowledge. Febrile serves as an up-to-date resource that can inform clinical practice while also acting as an effective teaching tool for ID educators. The sense of community and ability to role model positive aspects of a career in ID are important intangible benefits. The Febrile experience provides a glimpse into how to leverage the incredible potential of digital education to not only share ID knowledge but also excite learners ranging from early trainees to seasoned clinicians alike.

## Supplementary Material

ofae124_Supplementary_Data

## Supplementary Data

Supplementary materials are available at *Open Forum Infectious Diseases* online. Consisting of data provided by the authors to benefit the reader, the posted materials are not copyedited and are the sole responsibility of the authors, so questions or comments should be addressed to the corresponding author.
