# Supplementary material for: Impact of the Febrile Podcast and Learning Resource as an Infectious Diseases Education Platform
Source: Open Forum Infect Dis. 2024 Mar 4;11(4):ofae124. doi: 10.1093/ofid/ofae124 (PMC10977626; doi:10.1093/ofid/ofae124)
Supplement: ofae124_Supplementary_Data [file ofae124_supplementary_data.docx]

**Supplemental Table 1:  Febrile Contributors**

| **Contributor** | **Affiliation / Institution** | **City** | **State** | **Country** | **Episode(s)** | **# of episodes** | **Podcast Writing/**  **Review** | **Podcast Guest** | **Infographic Artist** | **Level of Training** |
| --- | --- | --- | --- | --- | --- | --- | --- | --- | --- | --- |
| Jeff Larnard | Beth Israel Deaconess Medical Center | Boston | MA | USA | 1 | 1 | X | X |  | Adult ID fellow |
| Wendy Stead | Beth Israel Deaconess Medical Center | Boston | MA | USA | 1,78 | 2 |  | X |  | Adult ID attending |
| Gerome Escota | Washington University, St. Louis | St. Louis | MO | USA | 2 | 1 |  | X |  | Adult ID attending |
| Nicole Theodoropoulos | University of Massachusetts | Worcester | MA | USA | 3 | 1 |  | X |  | Adult ID attending |
| Gabriella Lamb | Boston Children's Hospital | Boston | MA | USA | 4 | 1 |  | X |  | Pediatric ID attending |
| Anu Hazra | University of Chicago | Chicago | IL | USA | 5 | 1 |  | X |  | Adult ID attending |
| Jill Weatherhead | Baylor College of Medicine / Texas Children's Hospital | Houston | TX | USA | 6 | 1 |  | X |  | Med-Peds ID attending |
| Ruvandhi Nathavitharana | Beth Israel Deaconess Medical Center | Boston | MA | USA | 7 | 1 |  | X |  | Adult ID attending |
| Melanie Dubois | Boston Children's Hospital | Boston | MA | USA | 8 | 1 | X |  |  | Pediatric ID fellow |
| Thea Brennan-Krohn | Boston Children's Hospital | Boston | MA | USA | 8 | 1 |  | X |  | Pediatric ID attending |
| Tara Vijayan | University of California Los Angeles (UCLA) | Los Angeles | CA | USA | 9 | 1 |  | X |  | Adult ID attending |
| Juri Boguniewicz | Colorado Children's | Aurora | CO | USA | 10 | 1 |  | X |  | Pediatric ID attending |
| Rebecca Wallihan | Nationwide Children's Hospital | Columbus | OH | USA | 11 | 1 |  | X |  | Pediatric ID attending |
| Frances Ue | Cambridge Health Alliance | Cambridge | MA | USA | 12 | 1 | X | X |  | Hospitalist (Internal medicine) |
| Lou Ann Bruno-Murta | Cambridge Health Alliance | Cambridge | MA | USA | 12 | 1 |  | X |  | Adult ID attending |
| Darcy Wooten | University of California San Diego (UCSD) | San Diego | CA | USA | 13,54 | 2 | X | X |  | Adult ID attending |
| Meredith Clement | Louisiana State University | New Orleans | LA | USA | 14 | 1 |  | X |  | Adult ID attending |
| Rebecca Zash | Beth Israel Deaconess Medical Center | Boston | MA | USA | 15 | 1 |  | X |  | Adult ID attending |
| Leslie Enane | Indiana University / Riley Children's Hospital | Indianapolis | IN | USA | 16 | 1 |  | X |  | Pediatric ID attending |
| Kevin He | Beth Israel Deaconess Medical Center | Boston | MA | USA | 17,33 | 2 | X | X |  | Internal medicine resident |
| John Perfect | Duke University | Durham | NC | USA | 17 | 1 |  | X |  | Adult ID attending |
| Peter Krause | Yale University | New Haven | CT | USA | 18 | 1 |  | X |  | Adult ID attending |
| Lea Goren | University of Minnesota | Minneapolis | MN | USA | 19 | 1 | X | X | X | Medical student |
| Alice Lehman | University of Minnesota | Minneapolis | MN | USA | 19 | 1 | X | X | X | Med-Peds ID fellow |
| Beth Thielen | University of Minnesota | Minneapolis | MN | USA | 19 | 1 | X | X | X | Med-Peds ID attending |
| Ashka Patel | University of Maryland | College Park | MD | USA | 20 | 1 | X | X |  | Adult ID fellow |
| Jennifer Husson | University of Maryland | College Park | MD | USA | 20 | 1 |  | X |  | Adult ID attending |
| Zach Lorenz | Johns Hopkins Bayview Medical Center | Baltimore | MD | USA | 21,66 | 2 | X | X | X | Internal medicine resident |
| Khalil Ghanem | Johns Hopkins Bayview Medical Center | Baltimore | MD | USA | 21 | 1 |  | X |  | Adult ID attending |
| Saira Butt | Indiana University | Indianapolis | IN | USA | 22 | 1 |  | X |  | Adult ID attending |
| Jonathan Ryder | University of Nebraska | Omaha | NE | USA | 23,31,58,83,85 | 5 | X | X | X | Adult ID fellow |
| Evelyn Wu | Rutgers Robert Wood Johnson Medical School | New Brunswick | NJ | USA | 24 | 1 | X | X |  | Medical student |
| Lucy Marquez | Baylor College of Medicine / Texas Children's Hospital | Houston | TX | USA | 24 | 1 |  | X |  | Pediatric ID attending |
| Thomas Russo | University of Buffalo | Buffalo | NY | USA | 25 | 1 |  | X |  | Adult ID attending |
| Mo Bohlega | George Washington University | Washington | DC | USA | 26 | 1 | X | X |  | Internal medicine resident |
| Varun Phadke | Emory University | Atlanta | GA | USA | 26,67 | 2 |  | X |  | Adult ID attending |
| Jordan Mah | University of Calgary | Calgary |  | Canada | 27 | 1 | X | X |  | Adult ID fellow |
| Ilan Schwartz | University of Alberta | Alberta |  | Canada | 27 | 1 |  | X |  | Adult ID attending |
| Alejandra Mendoza | Mayo Clinic | Rochester | MN | USA | 29 | 1 | X | X |  | Adult ID fellow |
| John Wilson | Mayo Clinic | Rochester | MN | USA | 29 | 1 |  | X |  | Adult ID attending |
| Rebecca Kumar | MedStar Georgetown University Hospital | Washington | DC | USA | 30 | 1 |  | X |  | Adult ID attending |
| Nico Cortes-Penfield | University of Nebraska | Omaha | NE | USA | 31 | 1 |  | X |  | Adult ID attending |
| Arthur Jackson | Cork University Hospital / Mercy University Hospital | Cork |  | Ireland | 32,42 | 2 | X | X |  | Adult ID attending |
| Joseph Sassine | University of Oklahoma | Oklahoma City | OK | USA | 33,73,74 | 3 | X | X |  | Adult ID attending |
| Scott Crabtree | University of California Davis | Sacramento | CA | USA | 34 | 1 |  | X |  | Adult ID attending |
| Camille Kotton | Massachusetts General Hospita | Boston | MA | USA | 35 | 1 |  | X |  | Adult ID attending |
| Justin Penner | Children’s Hospital of Eastern Ontario | Ottawa |  | Canada | 36,41,82 | 3 | X | X |  | Pediatric ID attending |
| Nuria Sanchez-Clemente | St. George's University | London |  | UK | 37 | 1 |  | X |  | Paediatric ID registrar |
| Hermione Lyall | Imperial College | London |  | UK | 37 | 1 |  | X |  | Pediatric ID attending |
| Jeremey Walker | University of Alabama Birmingham | Birmingham | AL | USA | 38 | 1 |  | X | X | Adult ID attending |
| Ella Dzora | Sheffield Children's Hospital | Sheffield |  | UK | 39 | 1 |  | X |  | Paediatric registrar |
| Jason Brophy | Children's Hospital of Eastern Ontario | Ottawa |  | Canada | 39 | 1 |  | X |  | Pediatric ID attending |
| Julie Steinbrink | Duke University | Durham | NC | USA | 40 | 1 |  | X |  | Adult ID attending |
| Carsten Krueger | Children's Hospital of Eastern Ontario | Ottawa |  | Canada | 41 | 1 |  | X |  | Pediatric ID fellow |
| Clare Nourse | Queensland Children's Hospital | South Brisbane |  | Australia | 41 | 1 |  | X |  | Pediatric ID attending |
| Sarah May Johnson | Great Ormond St Hospital | London |  | UK | 43 | 1 |  | X |  | Paediatric ID registrar |
| Fani Ladomenou | Venizeleion General Hospital | Heraklion, Crete |  | Greece | 43 | 1 |  | X |  | Pediatric ID attending |
| Michael Cosimini | Oregon Health and Science University | Portland | OR | USA | 44 | 1 |  | X |  | Pediatrician |
| Cesar Burto | Jacobi Medical Center, Albert Einstein | Bronx | NY | USA | 45,64 | 2 | X | X | X | Internal medicine resident |
| Shweta Anjan | Jackson Memorial Hospital | Miami | FL | USA | 45 | 1 |  | X |  | Adult ID attending |
| Nathan Nolan | Washington University St. Louis | St. Louis | MO | USA | 46 | 1 | X | X |  | Adult ID attending |
| Raagini Jawa | Boston Medical Center | Boston | MA | USA | 46 | 1 |  | X |  | Adult ID attending |
| Shilpa Vashista | Mt Sinai | New York | NY | USA | 47,58 | 2 | X | X |  | Adult ID fellow |
| Christina Coyle | Jacobi Medical Center, Albert Einstein | Bronx | NY | USA | 47,67 | 2 |  | X |  | Adult ID attending |
| James Wilson | Rush University Medical Center | Chicago | IL | USA | 48 | 1 | X | X |  | Adult ID fellow |
| Ryan Maves | Wake Forest Baptist Medical Center | Winston-Salem | NC | USA | 48 | 1 |  | X |  | Adult ID attending |
| Kruti Yagnik | Cleveland Clinic Florida - Indian River Hospital | Vero Beach | FL | USA | 49 | 1 | X | X |  | Adult ID attending |
| Emily Niehaus | University of Utah | Salt Lake City | UT | USA | 50 | 1 | X | X |  | Internal medicine resident |
| Laura Certain | University of Utah | Salt Lake City | UT | USA | 50 | 1 |  | X |  | Adult ID attending |
| Jame McCrae | University of Edinburgh | Edinburgh, Scotland |  | UK | 51 | 1 | X | X |  | Adult ID & Clinical Pharmacology registrar |
| Callum Mutch | Lothian NHS | Edinburgh, Scotland |  | UK | 51 | 1 | X | X |  | Adult ID & Microbiology registrar |
| Saima Aslam | University of California San Diego | San Diego | CA | USA | 52 | 1 |  | X |  | Adult ID attending |
| Pratik Patel | Children's Healthcare of Atlanta; Emory University | Atlanta | GA | USA | 53,59 | 2 | X | X |  | Pediatric ID and Heme-Onc fellow |
| Joshua Wolf | St. Jude Children's Hospital | Memphis | TN | USA | 53 | 1 |  | X |  | Pediatric ID attending |
| James McClusky | University of Nebraska | Omaha | NE | USA | 55 | 1 | X | X |  | Med-Peds resident |
| Alice Sato | University of Nebraska | Omaha | NE | USA | 55 | 1 |  | X |  | Pediatric ID attending |
| Rachel Britt | University of Texas Medical Branch (UTMB) Health | Galveston | TX | USA | 56 | 1 |  | X |  | ID Pharmacist |
| Erin McCreary | University of Pittsburgh | Pittsburgh | PA | USA | 57 | 1 |  | X |  | ID Pharmacist |
| Jillian Hayes | Duke University | Durham | NC | USA | 57 | 1 |  | X |  | ID Pharmacist |
| Annie Jacobs | Carolinas Medical Center | Charlotte | NC | USA | 58,72 | 2 | X | X |  | Internal medicine resident |
| Robin Patel | Mayo Clinic | Rochester | MN | USA | 59 | 1 |  | X |  | Adult ID attending |
| Kevin Messacar | Children's Hospital Colorado | Auroro | CO | USA | 59 | 1 |  | X |  | Pediatric ID attending |
| Jeremey Steinbruck | Beaumont Royal Oak | Royal Oak | MI | USA | 60,61,62 | 3 | X | X |  | Adult ID fellow |
| Nick Gilpin | Beaumont Royal Oak | Royal Oak | MI | USA | 60,61,62 | 3 |  | X |  | Adult ID attending |
| Sam Schuiteman | University of Michigan | Ann Arbor | MI | USA | 63 | 1 | X | X |  | Internal medicine resident |
| Owen Albin | University of Michigan | Ann Arbor | MI | USA | 63 | 1 | X | X |  | Adult ID attending |
| Brad Cutrell | University of Texas Southwest (UTSW) | Dallas | TX | USA | 65 | 1 |  | X |  | Adult ID attending |
| Morgan Walker | Johns Hopkins Hospital | Baltimore | MD | USA | 66 | 1 |  | X |  | Adult ID and critical care fellow |
| Olivia Kates | Johns Hopkins Hospital | Baltimore | MD | USA | 66 | 1 |  | X |  | Adult ID attending |
| Katie Sharma | Emory University School of Medicine | Atlanta | GA | USA | 67 | 1 | X | X |  | Medical student |
| Amalia Aldredge | Emory University School of Medicine | Atlanta | GA | USA | 67 | 1 | X | X |  | Adult ID fellow |
| Navina Birk | Henry Ford Hospital | Detroit | MI | USA | 68 | 1 | X | X |  | Adult ID fellow |
| George Alangaden | Henry Ford Hospital | Detroit | MI | USA | 68 | 1 |  | X |  | Adult ID attending |
| Ralph Tayyar | Stanford University | Palo Alto | CA | USA | 69 | 1 | X | X |  | Adult ID fellow |
| Jessica Ferguson | Stanford University | Palo Alto | CA | USA | 69 | 1 | X | X |  | Adult ID attending |
| Anastasia Theodosiou | University of Southampton | Southampton |  | UK | 70 | 1 | X | X |  | Adult ID & Microbiology registrar |
| Chrissie Jones | University of Southampton | Southampton |  | UK | 70 | 1 |  | X |  | Pediatric ID attending |
| Annabelle De St Maurice | LA Department of Public Health | Los Angeles | CA | USA | 71 | 1 | X | X |  | Pediatric ID attending |
| Allison Bartlett | University of Chicago; Comer Children's | Chicago | IL | USA | 71 | 1 | X | X |  | Pediatric ID attending |
| Carol Vance | Advocate Aurora Children's Hospital; Interim Christ Medical Center | Chicago | IL | USA | 71 | 1 | X | X |  | MSN; Infection Prevention Director |
| Christopher Polk | Atrium Health Wake Forest | Charlotte | NC | USA | 72 | 1 |  | X |  | Adult ID attending |
| Rita Wilson | UTHealth; MD Anderson | Houston | TX | USA | 73,74 | 2 |  | X |  | Adult ID fellow |
| Luis Plaza | Advent Health | Orlando | FL | USA | 75 | 1 |  | X |  | Senior Medical Laboratory Scientists in Microbiology |
| Hawra Al-Lawati | Beth Israel Deaconess Medical Center | Boston | MA | USA | 3,6,76,77 | 4 | X | X | X | Adult ID fellow |
| Pranita Tamma | Johns Hopkins | Baltimore | MD | USA | 76 | 1 |  | X |  | Pediatric ID attending |
| Noah Rosenberg | Beth Israel Deaconess Medical Center | Boston | MA | USA | 78,83 | 2 | X | X |  | Internal medicine resident |
| Nick Palmeri | Beth Israel Deaconess Medical Center | Boston | MA | USA | 78 | 1 |  | X |  | Adult cardiology attending |
| Amedine Duret | Imperial College London; St. Mary's | London |  | UK | 79 | 1 |  | X |  | Paediatric ID registrar |
| Elizabeth Whittaker | Imperial College London; St. Mary's | London |  | UK | 79 | 1 |  | X |  | Pediatric ID attending |
| Fionnuala Ryan | Imperial College London | London |  | UK | 80 | 1 |  | X |  | Paediatric ID registrar |
| Alasdair Bamford | Great Ormond Street Hospital | London |  | UK | 80 | 1 |  | X |  | Pediatric ID attending |
| Felicity Fitzgerald | Imperial College London; St. Mary's | London |  | UK | 81 | 1 |  | X |  | Pediatric ID attending |
| Elizabeth O'Mahony | Imperial College London | London |  | UK | 81 | 1 |  | X |  | Paediatric registrar |
| Gunjan Mhapankar | Children's Hospital of Eastern Ontario | Ottawa |  | Canada | 82 | 1 |  | X |  | Pediatric ID fellow |
| Dylan Koundakjian | Emory University School of Medicine | Atlanta | GA | USA | 83 | 1 |  | X |  | Internal medicine resident |
| Alainna Jamal | University of Toronto | Toronto |  | Canada | 83 | 1 |  | X |  | Internal medicine resident |
| Carlyn Harris | Emory University School of Medicine | Atlanta | GA | USA | 83 | 1 |  | X |  | Medical student |
| Bismarck Bisono-Garcia | Mayo Clinic | Rochester | MN | USA | 83 | 1 |  | X |  | Adult ID fellow |
| Anais Ovalle | Kent with Care New England / Brown | Providence | RI | USA | 83 | 1 |  | X |  | Adult ID attending |
| Kailynn Jensen | University of Nebraska | Omaha | NE | USA | 83 | 1 |  | X |  | Medical student |
| Bill Wilson | University of North Carolina | Chapel Hill | NC | USA | 83 | 1 |  | X |  | ID Pharmacist |
| Raul Macias Gill | Harbor UCLA | Los Angeles | CA | USA | 83 | 1 |  | X |  | Adult ID attending |
| Rija Alvi | Henry Ford Hospital | Detroit | MI | USA | 83 | 1 |  | X |  | Adult ID fellow |
| Memar Ayalew | Walter Reed Hospital | Washington | DC | USA | 83 | 1 |  | X |  | ID Pharmacist |
| Radhika Sheth | Oregon Health Sciences University | Portland | OR | USA | 83 | 1 |  | X |  | Adult ID fellow |
| Julie England | University of Alabama Birmingham | Birmingham | AL | USA | 83 | 1 |  | X |  | Internal medicine resident |
| Christina Lin | Emory University School of Medicine | Atlanta | GA | USA | 83 | 1 |  | X |  | Internal medicine resident |
| Victoria Chu | University of California | San Francisco | CA | USA | 83 | 1 |  | X |  | Pediatric ID fellow |
| Diana Zhong | University of Pittsburgh | Pittsburgh | PA | USA | 83 | 1 |  | X |  | Adult ID attending |
| Abarna Pearl | Beth Israel Deaconess Medical Center | Boston | MA | USA | 83 | 1 |  | X |  | Adult ID fellow |
| Jaspreet Banga | Beth Israel Deaconess Medical Center | Boston | MA | USA | 83 | 1 |  | X |  | Adult ID fellow |
| Daniel Stanton | University of Texas Medical Branch (UTMB) Health | Galveston | TX | USA | 84 | 1 | X | X |  | Adult ID fellow |
| Christine Pho | University of Texas Southwest (UTSW) | Dallas | TX | USA | 84 | 1 |  | X |  | Medical student |
| Clinton White | University of Texas Medical Branch (UTMB) Health | Galveston | TX | USA | 84 | 1 |  | X |  | Adult ID attending |
| Rey Perez | Duke University | Durham | NC | USA | 85 | 1 | X | X |  | Adult ID fellow |
| Andrew Watkins | St. Dominic Jackson Memorial Hospital | Jackson | MS | USA | 85 | 1 | X | X |  | ID Pharmacist |
| Bashayer Alshehail | King Fahad Hospital; University in al Khobar | Riyadh |  | Saudi Arabia | 86 | 1 |  | X |  | ID Pharmacist |
| Efteraj Alhowity | King Salman Armed Forces Hospital | Tabuk |  | Saudi Arabia | 86 | 1 |  | X |  | Pediatric ID attending |
| Abdullah Almohaizeie | King Faisal Specialist Hospital | Riyadh |  | Saudi Arabia | 87 | 1 |  | X |  | ID Pharmacist |
| Fatimah Alshahrani | King Saud University Medical City | Riyadh |  | Saudi Arabia | 87 | 1 |  | X |  | Adult ID attending |
| Rabab Alghaithi | King Fahad Hospital | Riyadh |  | Saudi Arabia | 88 | 1 |  | X |  | Pediatric ID attending |
| Meshari Alabdullatif | BD | Riyadh |  | Saudi Arabia | 88 | 1 |  | X |  | Medical Microbiology |
| Scott James | University of Alabama Birmingham | Birmingham | AL | USA | 89 | 1 |  | X |  | Pediatric ID attending |
| Elise Merchant | Beth Israel Deaconess Medical Center | Boston | MA | USA | 5,14 | 2 | X |  | X | Adult ID fellow |
| Marcela Araujo de Oliveira Santana | Federal University of Uberlandia | Uberlandia |  | Brazil | 6,12,27 | 3 |  |  | X | Medical student |
| Kushal Vaishnani | Carolinas Medical Center | Charlotte | NC | USA | 7,17 | 2 | X |  |  | Hospitalist (Internal medicine) |

**Supplemental Table 2:  Number of survey responses per country and within the United States by state/district**

| **List of countries with responses** | Argentina | 2 |
| --- | --- | --- |
|  | Australia | 2 |
|  | Austria | 1 |
|  | Brazil | 1 |
|  | Canada | 4 |
|  | Chile | 1 |
|  | Colombia | 1 |
|  | Dominican Republic | 2 |
|  | France | 1 |
|  | Germany | 3 |
|  | Ghana | 1 |
|  | Guatemala | 1 |
|  | Hungary | 1 |
|  | India | 6 |
|  | Ireland | 4 |
|  | Israel | 3 |
|  | Italy | 1 |
|  | Latvia | 1 |
|  | Lebanon | 1 |
|  | Mexico | 5 |
|  | Pakistan | 2 |
|  | Saudi Arabia | 2 |
|  | South Africa | 2 |
|  | Spain | 2 |
|  | Switzerland | 2 |
|  | Thailand | 1 |
|  | Uganda | 1 |
|  | UK of Great Britain and Northern Ireland | 7 |
|  | USA | 159 |
|  | Zambia | 1 |
| **List of 50 States, DC, Puerto Rico with responses** | Alabama | 3 |
|  | Arizona | 2 |
|  | California | 13 |
|  | Colorado | 3 |
|  | Connecticut | 1 |
|  | DC | 5 |
|  | Florida | 6 |
|  | Georgia | 6 |
|  | Illinois | 2 |
|  | Indiana | 2 |
|  | Iowa | 2 |
|  | Kentucky | 3 |
|  | Louisiana | 1 |
|  | Maryland | 7 |
|  | Massachusetts | 12 |
|  | Michigan | 2 |
|  | Minnesota | 3 |
|  | Missouri | 4 |
|  | Nebraska | 8 |
|  | Nevada | 1 |
|  | New Hampshire | 1 |
|  | New Jersey | 5 |
|  | New Mexico | 2 |
|  | New York | 11 |
|  | North Carolina | 4 |
|  | Ohio | 6 |
|  | Oklahoma | 1 |
|  | Oregon | 1 |
|  | Pennsylvania | 11 |
|  | Rhode Island | 1 |
|  | South Carolina | 1 |
|  | South Dakota | 1 |
|  | Tennessee | 3 |
|  | Texas | 5 |
|  | Utah | 2 |
|  | Virginia | 2 |
|  | Washington | 5 |
|  | Wisconsin | 3 |

**Supplemental Table 3: Results from Figure 2 (Reported satisfaction with Febrile from survey respondents)**

| ***Question*** | **Responses** | | | |
| --- | --- | --- | --- | --- |
|  |  |  |  |  |
| *How satisfied are you with the episode length?* | *Not at all satisified* | *Slightly satisified* | *Moderately satisified* | *Extremely satisified* |
|  | 0 | 10 | 48 | 172 |
|  | 0.00% | 4.35% | 20.87% | 74.78% |
|  |  |  |  |  |
| *How satisfied are you with the quality of the website for Febrile?* | *Not at all satisified* | *Slightly satisified* | *Moderately satisified* | *Extremely satisified* |
|  | 0 | 2 | 30 | 134 |
|  | 0.00% | 1.20% | 18.07% | 80.72% |
|  |  |  |  |  |
| *How satisified are you with the quality of infographics of Febrile?* | *Not at all satisified* | *Slightly satisified* | *Moderately satisified* | *Extremely satisified* |
|  | 0 | 3 | 25 | 158 |
|  | 0.00% | 1.61% | 13.44% | 89.95% |
|  |  |  |  |  |
| *How satisfied are you with the audio quality of Febrile podcast?* | *Not at all satisified* | *Slightly satisified* | *Moderately satisified* | *Extremely satisified* |
|  | 0 | 4 | 27 | 198 |
|  | 0.00% | 1.75% | 11.79% | 86.46% |
|  |  |  |  |  |
| *How satisfied are you with the content organization of Febrile podcast?* | 0 | 4 | 8 | 187 |
|  | 0.00% | 1.75% | 16.59% | 81.66% |
|  |  |  |  |  |
| *How satisifed are you with the quality of guest discussants of Febrile podcast?* | 0 | 2 | 36 | 190 |
|  | 0.00% | 0.88% | 15.79% | 83.33% |
|  |  |  |  |  |
| *How satisfied are you with the quality of the host(s) of Febrile podcast?* | 0 | 2 | 21 | 207 |
|  | 0.00% | 0.87% | 9.13% | 90.00% |
|  |  |  |  |  |
| *Overall how much do you enjoy Febrile podcast?* | *Do not enjoy at all* | *Enjoy a little bit* | *Enjoy somewhat* | *Enjoy quite a bit* |
|  | 0 | 1 | 16 | 212 |
|  | 0.00% | 0.44% | 6.99% | 92.58% |
|  |  |  |  |  |
| *Is listening to Febrile podcast episodes an effective way to learn infectious diseases concepts?* | *Not at all effective* | *Slightly effective* | *Moderately effective* | *Extremely effective* |
|  | 1 | 4 | 67 | 158 |
|  | 0.43% | 1.74% | 29.13% | 68.70% |
|  |  |  |  |  |
| *How likely are you to recommend Febrile podcast to a colleague?* | *Do not recommend* | *Might recommend* | *Likely to recommend* | *Already recommended* |
|  | 0 | 2 | 35 | 190 |
|  | 0.00% | 0.88% | 15.42% | 83.70% |

**Supplemental Table 4: Results from Figure 3 (Perceived value and motivations for use of Febrile)**

| ***Question*** | **Responses** | | | |
| --- | --- | --- | --- | --- |
|  |  |  |  |  |
| *Why do you listen to Febrile podcast?* | *Yes* | *No* | *Yes* | *No* |
| *Enhancing ID core knowledge base* | 225 | 1 | 99.56% | 0.44% |
| *Clinical reasoning* | 208 | 14 | 93.69% | 6.31% |
| *To help me become a better educator of ID topics* | 191 | 28 | 87.21% | 12.79% |
| *Board exam preparation* | 96 | 105 | 47.76% | 54.24% |
| *Entertainment* | 135 | 67 | 66.83% | 33.17% |
| *Sense of community* | 117 | 83 | 58.50% | 41.50% |
| *Access to content experts or practics at other institutions* | 183 | 29 | 86.32% | 13.68% |
| *Keep up to date with the current literature or guidelines* | 209 | 10 | 95.43% | 4.57% |
| *Other* | 6 | 119 | 4.80% | 95.20% |
|  |  |  |  |  |
| *Has information you learned from a Febrile pocdast episode ever changed your clinical practice?* | *Yes* | *No* |  |  |
|  | 157 | 73 |  |  |
|  | 68.26% | 31.74% |  |  |
|  |  |  |  |  |
| *How has Febrile impacted your practice?* | *Yes* | *No* | *Yes* | *No* |
| *I considered an additional infection on my differential diagnosis* | 137 | 13 | 91.33% | 8.67% |
| *I selected a different diagnostic test* | 84 | 52 | 61.76%% | 38.24% |
| *I changed an antimicrobial I prescribed or recommended* | 65 | 70 | 48.15% | 51.85% |
| *I adjusted the duration of antimicrobial therapy* | 52 | 81 | 39.10% | 60.90% |
| *Other* | 4 | 74 | 5.13% | 94.87% |
|  |  |  |  |  |
| *Have you ever used Febrile podcast materials as a way to teach others?* | *Yes* | *No* |  |  |
|  | 117 | 113 |  |  |
|  | 50.87% | 49.13% |  |  |
|  |  |  |  |  |
| *How have you used Febrile to teach?* | *I have used* | *I have not used* | *I have used* | *I have not used* |
| *Assigned the podcast to learners to review a specific topic* | 67 | 43 | 60.91% | 39.09% |
| *Used podcast in flipped classroom teaching session* | 13 | 87 | 13.00% | 87.00% |
| *Used infographics to teach an ID topic on-the-fly or on rounds* | 87 | 24 | 78.38% | 21.62% |
| *Used infographics in a didactic presentation I prepared* | 57 | 48 | 54.29% | 45.71% |
| *Other* | 7 | 58 | 10.77% | 89.23% |
|  |  |  |  |  |
| *How has listening to Febrile podcast changed your perception of ID as a career?* | *I am already specializing in ID - favorable impact* | *I am already specializing in ID - no impact* | *I am already specializing in ID - negative impact* | *I am considering ID as a career - favorable impact* |
|  | 148 | 29 | 0 | 31 |
|  | 69.81% | 13.68% | 0.00% | 14.62% |

**Supplemental Material - Full Survey**

Febrile Utilization Survey

You are invited to participate in this survey as a listener of Febrile podcast.  The purpose of this survey is to understand how Febrile podcast is used and to seek your feedback for future content.

Your participation in this survey is voluntary, and you may choose not to participate.  You can skip any survey questions that you do not want to answer. If you do decide to participate, you may withdraw at any time. This survey is anonymous, and no one will be able to link your answers back to you. Responses will be reported in aggregated format.

The survey is brief and should take 10 minutes to complete.  Please contact Sara Dong at swdong@bidmc.harvard.edu with questions about this survey. If you want to participate, click the Agree button to start the survey

o Agree  (1)

Q1 How many episodes of Febrile podcast have you listened to?

o ≤10  (1)

o 11 to 20  (2)

o ≥21  (3)

Q2 Do you ever listen to an episode more than once?

o Yes  (1)

o No  (2)

Q3 How satisfied are you with the episode length (30-60 minutes)?

o Not at all satisfied  (1)

o Slightly satisfied  (2)

o Moderately satisfied  (3)

o Extremely satisfied  (5)

Q4 Have you visited the febrilepodcast.com website?

o Yes  (1)

o No  (2)

Display This Question:

If Have you visited the febrilepodcast.com website? = Yes

Q6 How many times have you accessed the website?

o 1 to 2 times  (1)

o 3 to 5 times  (2)

o >5 times  (3)

 Display This Question:

If Have you visited the febrilepodcast.com website? = Yes

Q7 Did you look at the "Consult Notes" for an episode?

o Yes  (1)

o No  (2)

Display This Question:

If Have you visited the febrilepodcast.com website? = Yes

Q8 How satisfied are you with the quality of the website for Febrile?

o Not at all satisfied  (1)

o Slightly satisfied  (2)

o Moderately satisfied  (3)

o Extremely satisfied  (5)

Q9 Have you seen a Febrile podcast related infographic?

o Yes  (1)

o No  (2)

Display This Question:

If Have you seen a Febrile podcast related infographic? = Yes

Q10 Where have you seen Febrile infographics?

|  | Yes (1) | No (2) |
| --- | --- | --- |
| On Twitter (1) | o | o |
| On Instagram (2) | o | o |
| On febrilepodcast.com website (3) | o | o |
| Used in teaching materials I saw (4) | o | o |
| Other (5) | o | o |

Display This Question:

If Where have you seen Febrile infographics? = Other [ Yes ]

Q11 Where else have you seen Febrile infographics?

________________________________________________________________

Display This Question:

If Have you seen a Febrile podcast related infographic? = Yes

Q12 How satisfied are you with the quality of the infographics of Febrile?

o Not at all satisfied  (1)

o Slightly satisfied  (2)

o Moderately satisfied  (3)

o Extremely satisfied  (5)

Q13 Have you ever used Febrile podcast materials as a way to teach others?

o Yes  (1)

o No  (2)

 Display This Question:

If Have you ever used Febrile podcast materials as a way to teach others? = Yes

Q14 How have you used Febrile to teach?

|  | I have used (1) | I have not used (2) |
| --- | --- | --- |
| Assigned the podcast to learners to review a specific topic (1) | o | o |
| Used podcast in flipped classroom teaching session (2) | o | o |
| Used infographics to teach an ID topic on-the-fly or on rounds (3) | o | o |
| Used infographics in a didactic presentation I prepared (4) | o | o |
| Other (5) | o | o |

Display This Question:

If How have you used Febrile to teach? = Other [ I have used ]

Q15 You selected other.  How else have you used Febrile podcast to teach?

________________________________________________________________

Q16 How did you first learn about Febrile podcast?

o Social media (such as Twitter or Instagram)  (1)

o A colleague directly recommended to me  (2)

o Search engine  (3)

o iTunes or podcast player suggestion  (4)

o ID Fellows Cup or ID Fellows Network  (5)

o Other: specify  (6)

Display This Question:

If How did you first learn about Febrile podcast? = Other: specify

Q17 You selected other. How did you first learn about Febrile?

________________________________________________________________

Q18 Overall how much do you enjoy Febrile podcast?

o Do not enjoy at all  (1)

o Enjoy a little bit  (2)

o Enjoy somewhat  (3)

o Enjoy quite a bit  (4)

Q20 How satisfied are you with the audio quality of Febrile podcast?

o Not at all satisfied  (1)

o Slightly satisfied  (2)

o Moderately satisfied  (3)

o Extremely satisfied  (5)

Q21 How satisfied are you with the content organization of Febrile podcast?

o Not at all satisfied  (1)

o Slightly satisfied  (2)

o Moderately satisfied  (3)

o Extremely satisfied  (5)

Q22 How satisfied are you with the quality of guest discussants of Febrile podcast?

o Not at all satisfied  (1)

o Slightly satisfied  (2)

o Moderately satisfied  (3)

o Extremely satisfied  (5)

Q24 How satisfied are you with the quality of the host(s) of Febrile podcast?

o Not at all satisfied  (1)

o Slightly satisfied  (2)

o Moderately satisfied  (3)

o Extremely satisfied  (5)

Q25 Why do you listen to Febrile podcast?

|  | Yes (1) | No (2) |
| --- | --- | --- |
| Enhancing ID core knowledge base (1) | o | o |
| Clinical reasoning (3) | o | o |
| To help me become a better educator of ID topics (4) | o | o |
| Board exam preparation (5) | o | o |
| Entertainment (6) | o | o |
| Sense of community (7) | o | o |
| Access to content experts or practices at other institutions (8) | o | o |
| Keep up to date with current literature or guidelines (10) | o | o |
| Other (13) | o | o |

Display This Question:

If Why do you listen to Febrile podcast? = Other [ Yes ]

Q26 You selected other. Why do you listen to Febrile podcast?

________________________________________________________________

Q27 Is listening to Febrile podcast episodes an effective way to learn infectious diseases concepts?

o Not at all effective  (1)

o Slightly effective  (2)

o Moderately effective  (3)

o Extremely effective  (5)

Q28 Has information you learned from a Febrile podcast episode ever changed your clinical practice?

o Yes  (1)

o No  (2)

Display This Question:

If Has information you learned from a Febrile podcast episode ever changed your clinical practice? = Yes

Q29 How has Febrile impacted your practice?

|  | Yes (1) | No (2) |
| --- | --- | --- |
| I considered an additional infection on my differential diagnosis (1) | o | o |
| I selected a different diagnostic test (2) | o | o |
| I changed an antimicrobial I prescribed or recommended (3) | o | o |
| I adjusted the duration of antimicrobial therapy (4) | o | o |
| Other (5) | o | o |

Display This Question:

If How has Febrile impacted your practice? = Other [ Yes ]

Q30 You selected other. How has Febrile impacted your practice?

________________________________________________________________

Q31 How likely are you to recommend Febrile podcast to a colleague?

o Do not recommend  (1)

o Might recommend  (2)

o Likely to recommend  (3)

o Already recommended  (4)

Q32 How has listening to Febrile podcast changed your perception of ID as a career?

o I am already specializing in ID - favorable impact  (1)

o I am already specializing in ID - no impact  (2)

o I am already specializing in ID - negative impact  (3)

o I am considering ID as a career - favorable impact  (4)

o I am considering ID as a career - no impact  (5)

o I am consider ID as a career - negative impact  (6)

o N/A  (7)

Q33 Any additional comments on how Febrile has impacted your perception of ID as a career?

________________________________________________________________

Q34 What are the strengths of Febrile podcast?

________________________________________________________________

Q35 What could be improved with Febrile podcast materials?  This could be related to audio podcast, website, or written or infographic materials.

________________________________________________________________

Q36 Do you have requests or recommendations for future ID topics or guests for Febrile?

________________________________________________________________

Q41 Are you an infectious diseases fellow in training? (physician such as MD, DO, MBBS)

o Yes  (1)

o No  (2)

Display This Question:

If Are you an infectious diseases fellow in training? (physician such as MD, DO, MBBS) = Yes

Q42 Are you an adult and/or pediatric ID fellow?

o Adult ID fellow  (1)

o Pediatric ID fellow  (2)

o Combined adult and pediatric fellow  (3)

Display This Question:

If Are you an infectious diseases fellow in training? (physician such as MD, DO, MBBS) = Yes

Q43 What is your current year of fellowship training?

o First year  (1)

o Second year  (2)

o Third year  (3)

o Fourth year or more  (4)

Display This Question:

If Are you an infectious diseases fellow in training? (physician such as MD, DO, MBBS) = No

Q44 What is your professional role?

o Medical student  (1)

o Other type of student  (2)

o Medical resident or registrar  (3)

o Fellow-in-training, non-infectious diseases  (4)

o Faculty physician (MD, DO, MBBS), infectious diseases  (5)

o Faculty physician, non-infectious diseases  (6)

o Advanced practice provider or APP student (NP, PA)  (7)

o Pharmacy resident  (8)

o Pharmacist  (9)

o Microbiologist or lab technician  (10)

o Nurse  (11)

o Other healthcare professional  (12)

o I am not a healthcare professional  (13)

Display This Question:

If What is your professional role? = Medical student

Q45 What year of medical school?

o First  (1)

o Second  (2)

o Third  (3)

o Fourth or more  (4)

Display This Question:

If What is your professional role? = Other type of student

Q54 Which type of student?

________________________________________________________________

Display This Question:

If What is your professional role? = Medical resident or registrar

Or What is your professional role? = Fellow-in-training, non-infectious diseases

Or What is your professional role? = Faculty physician, non-infectious diseases

Q46 What is your specialty?

________________________________________________________________

Display This Question:

If What is your professional role? = Faculty physician (MD, DO, MBBS), infectious diseases

Q47 Do you specialize in adult, pediatric, or combined Med-Peds ID?

o Adult  (1)

o Pediatric  (2)

o Adult & Pediatric  (3)

Display This Question:

If What is your professional role? = Faculty physician (MD, DO, MBBS), infectious diseases

Or What is your professional role? = Faculty physician, non-infectious diseases

Q48 How many years have you been in practice?

o <1 year  (1)

o 1-2 years  (2)

o 3-5 years  (3)

o >5 years  (4)

Display This Question:

If What is your professional role? = Pharmacy resident

Q49 Which year are you in?

o PGY1  (1)

o PGY2  (2)

|  |
| --- |

Q1 In which country do you currently reside?

▼ Afghanistan (1) ... Zimbabwe (1357)

Display This Question:

If List of Countries = United States of America

Q1 In which state do you currently reside?

▼ Alabama (1) ... I do not reside in the United States (53)

 Q57 What is your age?

o 25 and under  (2)

o 26 to 35  (3)

o 36 to 45  (4)

o 46 to 55  (5)

o 56 and older  (6)

Display This Question:

If What is your professional role? = Other healthcare professional

And What is your professional role? = I am not a healthcare professional

Q58 You selected other.  What is your occupation?

________________________________________________________________

Q59 What is your practice setting?

o Academic  (1)

o Community  (2)

o Industry  (3)

o Military  (4)

o Other  (5)

Display This Question:

If What is your practice setting? = Other

Q60 You selected other.  What is your practice setting?

________________________________________________________________

Q52 Would you be interested in participating in an interview or focus group aimed at understanding utilization and satisfaction of Febrile podcast?

o Yes  (1)

o No  (2)

Q53 If you selected yes, please provide contact email.  Responses will be de-identified and collected in aggregate form.
